# Retweets as a Predictor of Relationships among Users on Social Media

**DOI:** 10.1371/journal.pone.0170279

**Published:** 2017-01-20

**Authors:** Sho Tsugawa, Kosuke Kito

**Affiliations:** 1 Faculty of Engineering, Information and Systems, University of Tsukuba, Ibaraki, Japan; 2 School of Media Arts, Science and Technology, University of Tsukuba, Ibaraki, Japan; Centre de physique theorique, FRANCE

## Abstract

Link prediction is the problem of detecting missing links or predicting future link formation in a network. Application of link prediction to social media, such as Twitter and Facebook, is useful both for developing novel services and for sociological analyses. While most existing research on link prediction uses only the social network topology for the prediction, in social media, records of user activities such as posting, replying, and reposting are available. These records are expected to reflect user interest, and so incorporating them should improve link prediction. However, research into link prediction using the records of user activities is still in its infancy, and the effectiveness of such records for link prediction has not been fully explored. In this study, we focus in particular on records of reposting as a promising source that could be useful for link prediction, and investigate their effectiveness for link prediction on the popular social media platform Twitter. Our results show that (1) the prediction accuracy of techniques using reposting records is higher than that of popular topology-based techniques such as common neighbors and resource allocation for actively retweeting users, (2) the accuracy of link prediction techniques that use network topology alone can be improved by incorporating reposting records.

## Introduction

Link prediction is a fundamental problem in social network research, and has been actively studied [1–10]. Typically, link prediction is the problem of detecting missing links or predicting future link formation in a network by utilizing a given network topology [1]. In the literature, several link prediction techniques have been proposed, and these techniques have been applied to several types of social networks [2, 5, 6, 9–12]. Link prediction techniques have a broad range of application domains, and are expected to be utilized for recommendation [1], anomaly detection [13], network modeling [14], missing link detection [7], evaluation of network evolution mechanisms [15], reconstruction of networks [16], and classification of partially labeled networks [17, 18].

Application of link prediction to social media, such as Twitter and Facebook, is useful both for developing novel services and for sociological analyses. Link prediction techniques can be used for predicting future link formation, which is expected to be useful for user recommendation in social media. Moreover, link prediction applied to social networks on social media can help with research in the area of *computational social sciences* [19]. While social network data are a powerful source for computational social science research, they typically contain errors such as missing links and false links [20, 21]. Link prediction techniques can reduce such errors by predicting missing links or detecting false links in the dataset [7].

While most existing research on link prediction uses only the social network topology for the prediction [1–3, 7], in social media, records of user activities such as posting, replying, and reposting are available. These records are expected to reflect user interest, and so incorporating them should improve link prediction.

We focus in particular on records of reposting, which is known as *retweeting* in Twitter, as a promising source that could be useful for link prediction. Recent work has found that the information flow generated by user reposting is an important mechanism of link creation in social media [22, 23]. Moreover, Zhu *et al.* [24] and Li *et al.* [25] have used reposting records in social media for link prediction. However, research into link prediction using the records of reposting is still in its infancy, and the effectiveness of such records for link prediction has not been fully explored.

In this study, we extensively investigate the effectiveness of user records of reposting for link prediction in social media. We focus in particular on the popular social media platform Twitter and examine how records of retweets are useful for predicting links in the follower network. We perform experiments of both future link prediction and missing link detection, and investigate the prediction accuracy of techniques using the records of retweets. Our main contributions are summarized as follows.

- We extensively investigate the effectiveness of the records of reposting for link prediction, and show that the records of reposting are a promising source for link prediction. We show that the prediction accuracy of retweet-based techniques is higher than that of popular topology-based techniques such as common neighbors and resource allocation for actively retweeting users.
- We reveal useful features for link prediction obtained from the records of reposting. We propose using two features, which we call *retweet views* and *retweet posts*. While existing research using reposting records for link prediction focuses on *retweet views* [24, 25], we show that *retweet posts* is more effective than *retweet views* for link prediction.
- We demonstrate that combining reposting records and network topology can improve the accuracy of link prediction. We show that the accuracy of link prediction techniques that use network topology alone can be improved by incorporating reposting records.

## Related Work

In the literature, several link prediction techniques have been proposed. Many researchers have used an unsupervised approach for link prediction [1, 2, 7, 10–12, 25]. Unsupervised link prediction techniques estimate the likelihood of link formation (i.e., link prediction score) between two nodes by using knowledge about the characteristics of real networks. For instance, one of the most popular link prediction techniques, the common neighbors method (CN), estimates the likelihood of link formation based on the idea that the existence of many common adjacent nodes between two nodes implies a high probability of link formation between them [11]. Existing techniques aim to predict link formation or to detect missing links from only the topological structure of social networks [1, 2, 7, 10–12, 25]. In contrast, we focus on social networks in social media systems, and examine the effectiveness of examining records of user activity for link prediction.

Supervised approaches for link prediction have also been proposed [8, 26, 27]. Supervised approaches construct a classifier that can predict whether a link exists or not between two nodes, by using several features obtained from the network topology. Here, we use an unsupervised approach rather than a supervised approach because the link prediction score from unsupervised techniques can be also used as an effective feature for supervised link prediction.

Recently, link prediction using heterogeneous networks has been studied [28–31]. For instance, Pujari *et al.* [31] and Sunet *et al.* [30] studied the co-authorship link prediction problem using heterogeneous bibliographic networks such as networks representing co-authorship, co-venue, and co-citing relationships. These studies show that using multiple metrics obtained from heterogeneous networks greatly improves the performance of both unsupervised and supervised link prediction compared with using only a single metric obtained from a network. Reposting relationships among social media users can be regarded as a network. Therefore, we expect that combining reposting networks and follower networks in social media is an effective approach for link prediction.

Weng *et al.* [22] and Myers *et al.* [23] showed that information flow generated by reposting is a major factor of link formation in social media, demonstrating the potential of records of reposting for link prediction. The main objective of these studies was analysis of network evolution, and therefore the prediction accuracy of using the records of reposting for link prediction was not shown in these studies.

There exist notable exceptions of using reposting records for link prediction tasks in the literature [24, 25]. Zhu *et al.* [24] and Li *et al.* [25] proposed techniques that use reposting records for link prediction in which the likelihood of link formation is estimated based on *retweet views*. Namely, the likelihood of link formation is estimated by assuming that as user *i* see more tweets that are posted by user *j*, the probability that user *i* will follow user *j* increases. Our study builds on this work and contributes to improving techniques for link prediction by using reposting records. While previous studies use data about social media users in the United States [24] or China [25], we examine the effectiveness of link prediction techniques using reposting records for Japanese social media users, and validate the generalizability of the existing work. Moreover, extending the idea behind the existing work, we propose estimation of the likelihood of link formation based on *retweet posts*, and examine its effectiveness. Link prediction based on retweet posts is based on the idea that as user *i* retweets more tweets that are posted by user *j*, the probability that user *i* will follow user *j* increases.

## Methodology

### Problem formulation and accuracy measures

Let *G*_*o*_ = (*V*, *E*_*o*_) and *G*_*t*_ = (*V*, *E*_*t*_) be directed unweighted networks where network *G*_*o*_ represents the observed network and network *G*_*t*_ represents the future network or true network in which we would like to predict the links.

In the link prediction problem, for each node pair (*i*, *j*) ∉ *E*_*o*_, we predict whether (*i*, *j*) ∈ *E*_*t*_ or (*i*, *j*) ∉ *E*_*t*_ using the observed network *G*_*o*_. Here, in addition to network *G*_*o*_, the records of retweets posted during a specific period are also available for link prediction.

For each node pair (*i*, *j*) ∉ *E*_*o*_, we calculate the link prediction score *l*(*i*, *j*), which estimates the likelihood of link formation or the existence of a link from node *i* to node *j*. The link prediction score *l*(*i*, *j*) is obtained from the observed network *G*_*o*_ and the records of retweets.

To evaluate prediction accuracy, we use precision and recall following prior work [9, 25, 26, 32]. Although receiver operating characteristic (ROC) curve and area under the ROC curve (AUC) are also widely used for evaluating link prediction, we adopt precision and recall since as discussed in [33, 34], precision and recall provide a more discriminative view of classification performance in extremely imbalanced scenario such as link prediction. We extract node pairs where the link prediction scores *l*(*i*, *j*) meet or exceed a threshold *T*, and then calculate the precision *P* and recall *R* as defined by the following equations.

$$P=\frac{TP}{TP+FP}$$ (1)

$$R=\frac{TP}{TP+FN}$$ (2)

where TP (true positive), TN (true negative), FP (false positive), and FN (false negative) represent the respective numbers of node pairs (*i*, *j*) satisfying the corresponding conditions in Table 1. Precision evaluates the correctness whereas recall evaluates the completeness of link prediction. Generally, there is a tradeoff between precision and recall such that a larger threshold *T* increases precision and decreases recall.

**Table 1:** Definitions of true positive (TP), true negative (TN), false positive (FP), and false negative (FN): TP, TN, FP, and FN are the numbers of node pairs (*i*, *j*) satisfying the conditions shown in this table.

	(*i*, *j*) ∉ *E*_*o*_ and (*i*, *j*)∈*E*_*t*_	(*i*, *j*) ∉ *E*_*o*_ and (*i*, *j*) ∉ *E*_*t*_
*l*(*i*, *j*) ≥ *T*	TP	FP
*l*(*i*, *j*) < *T*	FN	TN

### Prediction scores

In this study, we use two link prediction techniques based on network topology and two techniques based on the records of retweets. Among techniques based on network topology, we use two popular neighborhood-based link prediction techniques: CN [11] and resource allocation (RA) [10]. Since Weng [22]*et al.* reported that approximately 85% of new links are created by following *a friend of a friend* on social media, we expect that neighborhood-based techniques are effective for link prediction on Twitter. In what follows, we introduce the link prediction score of each technique as well as the link prediction score of combining multiple link prediction techniques.

#### Common neighbors

The first link prediction technique based on network topology is CN, which predicts link formation based on the idea that the existence of many common adjacent nodes between two nodes implies a high probability of new link formation between those two nodes [11]. In CN [11], *l*(*i*, *j*), an estimate of the likelihood that node *i* will follow node *j*, is given by

$$l_{CN}\left(i,j\right)=\left|\Gamma_{\text{OUT}}\left(i\right)\cap \Gamma_{\text{IN}}\left(j\right)\right|$$ (3)

where $\Gamma_{\text{OUT}}\left(i\right)$ is a set of nodes that are followed by node *i*, and $\Gamma_{\text{IN}}\left(j\right)$ is a set of nodes that are following node *j*.

#### Resource allocation

The second link prediction technique based on network topology is called resource allocation (RA). Resource allocation predicts new link formation based on the idea that many common adjacent nodes with small degree between two nodes implies a high probability of new link formation between the nodes [10]. Similarly to CN, RA predicts new link formation on the basis of the number of common adjacent nodes, but assign a weight to *l*(*i*, *j*) based on the degree of common adjacent nodes. In RA [10], *l*(*i*, *j*), an estimate of the likelihood that node *i* will follow node *j*, is given by

$$l_{RA}\left(i,j\right)=\sum_{k\in |\Gamma_{\text{OUT}}\left(i\right)\cap \Gamma_{\text{IN}}\left(j\right)|}\frac{1}{|\Gamma_{\text{IN}}\left(k\right)|}.$$ (4)

#### Retweet views

**Fig 1:**
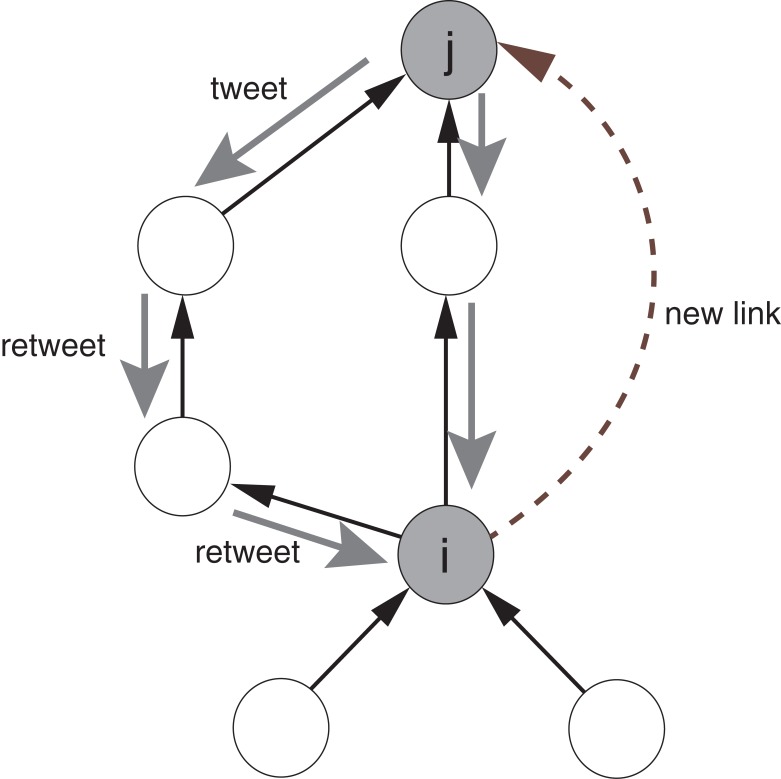
Assumptions of link prediction based on retweet views. There exists a positive correlation between the number of times that user *i* sees *j*’s retweets of posts by users that *i* is following and the probability that *i* follows user *j*.

The first link prediction technique based on the records of retweets predicts user link formation from the number of retweet views of the user. We call this technique RTV (ReTweet Views). RTV predicts link formation assuming that as user *i* see more tweets that are posted by user *j*, the probability that user *i* will follow user *j* increases (Fig 1). This assumption is the same as in existing work [24, 25]. In RTV, *l*(*i*, *j*), an estimate of the likelihood that node *i* will follow node *j*, is given by the following equation [25].

$$l_{RTV}\left(i,j\right)=\sum_{k\in \Gamma_{\text{OUT}}\left(i\right)}n_{k,j}$$ (5)

where *n*_*k*,*j*_ is the number of retweets of user *j*’s original tweets by user *k* during a fixed time interval.

#### Retweet posts

**Fig 2:**
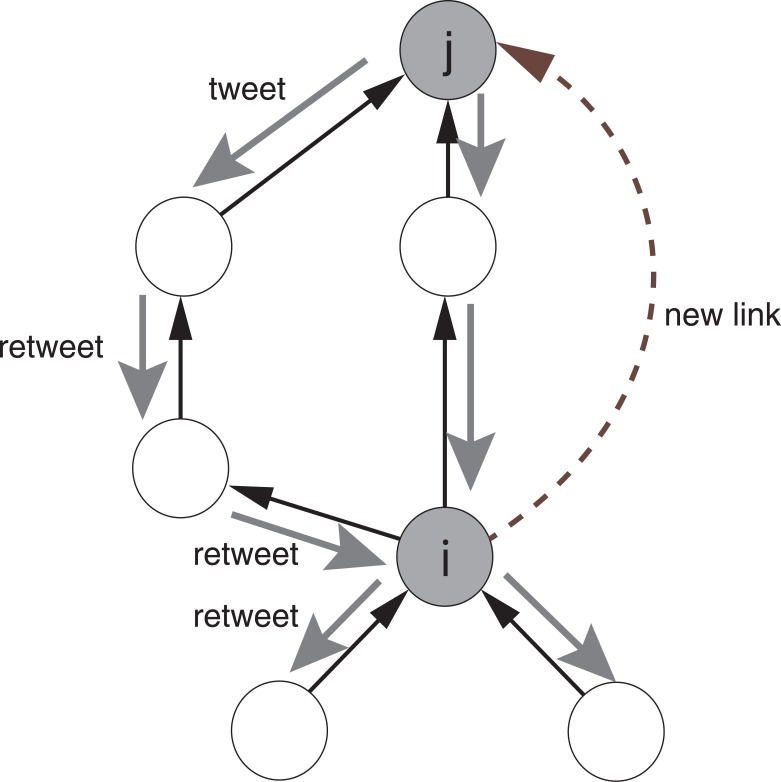
Assumptions of link prediction based on retweet posts. There exists a positive correlation between the number of retweets of user *j*’s tweets by user *i* and the probability that *i* will follow *j*.

The second link prediction technique based on the records of retweets predicts user link formation from the number of retweet *posts* by the user rather than retweet views. We call this technique RTP (ReTweet Posts). RTP extends the idea of RTV, and predicts link formation assuming that as user *i* retweets more tweets that are posted by user *j*, the probability that user *i* will follow user *j* increases (Fig 2). We expect that frequent retweeting of user *j*’s tweets by user *i* implies user *i* is interested in user *j*. In RTP, *l*(*i*, *j*), an estimate of the likelihood that node *i* will follow node *j*, is given by the following equation.

$$l_{RTP}\left(i,j\right)=n_{i,j}$$ (6)

#### Simple combination

In this study, we also examine the effectiveness of combining multiple link prediction scores. The link prediction score given by combining link prediction scores *l*_*A*_(*i*, *j*) and *l*_*B*_(*i*, *j*) is given by

$$l_{MIX}\left(i,j\right)=w\frac{l_{A}\left(i,j\right)}{\sigma_{A}}+\left(1-w\right)\frac{l_{B}\left(i,j\right)}{\sigma_{B}}$$ (7)

where *σ*_*A*_ and *σ*_*B*_ are the standard deviations of link prediction score *l*_*A*_(*i*, *j*) and *l*_*B*_(*i*, *j*), respectively, and *w* is a weighting parameter.

#### Rank aggregation

We also use the rank aggregation [35] for combining multiple link prediction scores. Among two rank aggregation methods proposed in [35], we chose the Borda score as a link prediction score considering their computational costs. The link prediction score when aggregating two link prediction technique A and B is given by

$$l_{rank}\left(i,j\right)=\beta_{A}\left(M-L_{A}\left(i,j\right)\right)+\beta_{B}\left(M-L_{B}\left(i,j\right)\right)$$ (8)

where *M* is the number of node pairs (*i*, *j*) ∉ *E*_*o*_, and *L*_*A*_(*i*, *j*) and *L*_*B*_(*i*, *j*) are the rankings of node pair (*i*, *j*) when node pairs are ranked in descending order of link prediction score *l*_*A*_(*i*, *j*) and *l*_*B*_(*i*, *j*), respectively. *β*_*A*_ and *β*_*B*_ are weighting parameters. In [35], optimal weighting parameters are determined by using training data since Ref. [35] uses supervised approach. In contrast, since we use unsupervised approach, we investigate the prediction accuracy of rank aggregation when changing these weighting parameters.

### Dataset and experimental setup

To investigate the effectiveness of records of retweets for link prediction, we collected large-scale data of both retweets and following relationships on Twitter. Since the usage patterns of Twitter users differ across languages [36], we used tweets from Japanese Twitter users to focus on users with the same culture and to eliminate the effects of different time zones.

Using the Twitter application programming interface (API) (https://dev.twitter.com/overview/api), we collected Japanese retweets posted in the period from December 11, 2013, to January 31, 2014. We used the Search API in Twitter REST API v1.1, and collected Japanese tweets using the query q = RT, lang = ja. From obtained records (each record corresponds to one tweet), we extracted records that had the *retweeted_status* field set to obtain retweets. We obtained 406,424,307 retweets during the period.

We determined target users for link prediction from the users who post the collected retweets. For the purpose of the experiment, we extracted active users who performed retweeting frequently during the period of December 11 to 17, 2013, by the following procedure. We first counted the number of retweets of the original tweets posted during the period, then extracted the original tweets whose number of retweets was between 10 and 100. We then extracted users who retweeted 10 or more of these tweets, which gave 356,453 users.

We next obtained snapshots of the social networks of the 356,453 users as of early January 2014, and February 2014. We obtained the followers and followees of the 356,453 users by using the Twitter API in early January 2014 for the period from January 1 to 11, 2014, and in early February 2014 for the period from February 1 to 10, 2014. We refer to the social network of the target users in early January 2014 and February 2014 as *G*_1_ = (*V*, *E*_1_) and *G*_2_ = (*V*, *E*_2_), respectively, where *V* is a set of nodes representing the target users and *E*_1_ and *E*_2_ are sets of links representing following relationships originating from and pointing to the target users as of early January 2014 and February 2014, respectively.

We investigate the effectiveness of retweets for the two-types of link prediction tasks: *future link prediction* and *missing link detection*. In the future link prediction task, the observed network *G*_*o*_ is *G*_1_ and the future network *G*_*t*_ is *G*_2_. For the missing link detection task, we synthetically generate a network with missing links by deleting the links in *G*_2_. Specifically, we obtain a directed unweighted network $G_{2}^{\prime }=\left(V,E_{2}^{\prime }\right)$ by deleting edges (*i*, *j*) ∈ *E*_2_(*i*, *j* ∈ *V*) with a probability 0.05. In the missing link detection task, the observed network *G*_*o*_ is $G_{2}^{\prime }$ and the true network *G*_*t*_ is *G*_2_. For both tasks, we used the observed network *G*_*t*_ and the records of retweets posted during January 1, 2014, to January 31, 2014. Namely, link prediction scores were obtained from *G*_*t*_ and retweets during the period. We randomly selected 30,000 users from the 356,453 users, and the links of the 30,000 users were predicted. More specifically, let *V*′ be the set of 30,000 randomly selected users. We calculated the link prediction score *l*(*i*, *j*) for *i* ∈ *V*′ and *j* ∈ *V*, and then determined the precision and recall. Several statistics about the dataset used in this study are shown in Table 2. The largest weakly connected component contains 99.5% of nodes in *G*_1_, *G*_2_, and $G_{2}^{\prime }$. There exist 1,640, 1,550, and 1,649 weakly connected components in *G*_1_, *G*_2_, and $G_{2}^{\prime }$ respectively.

**Table 2:** Statistics of the dataset used in this study.

Number of users with links to be predicted	30,000
Number of nodes in *G*_1_ and *G*_2_	356,453
Number of links originating from the target users in *G*_1_	15,280,387
Number of links originating from the target users in *G*_2_	15,932,773
Number of retweets per user during January 2014	273.3

## Results

### Correlation analysis

**Fig 3:**
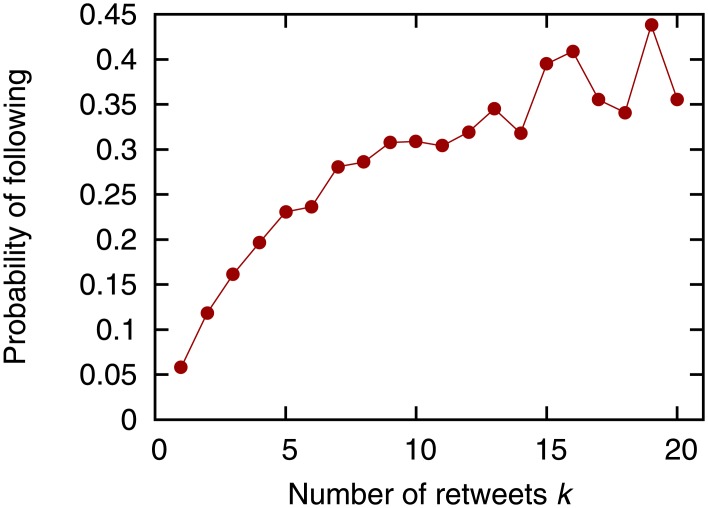
The number of retweets vs. the probability of following.

**Fig 4:**
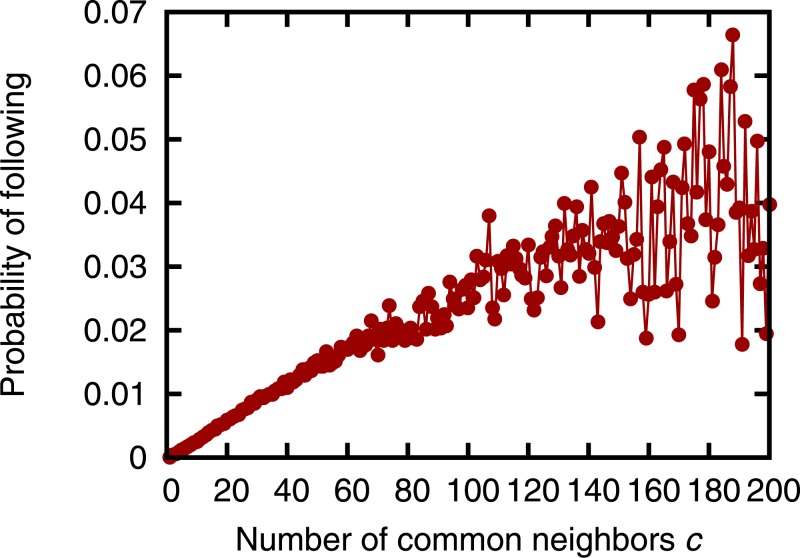
The number of common neighbors vs. the probability of following.

Before evaluating the prediction accuracy, we first examine the correlation between link formation and retweets. Following Li *et al.* [25], we investigate the probability that user *i* will follow user *j* when user *i* retweets exactly *k* of user *j*’s tweets. Specifically, we obtain the ratio of the number of node pairs (*i*, *j*) ∉ *E*_1_, where *n*_*i*,*j*_ = *k* and (*i*, *j*)∈*E*_2_, to the number of node pairs (*i*, *j*) ∉ *E*_1_, where *n*_*i*,*j*_ = *k*. We then investigate the relation between the number of retweets *k* and the obtained ratio (Fig 3). Note that Fig 3 does not include the results where *k* > 20 since the fraction of node pairs (*i*, *j*) where *n*_*i*,*j*_ > 20 is only about 0.2%. Fig 3 shows that there is a positive correlation between the number of retweets and the probability of following. This result supports our hypothesis that as user *i* retweets more tweets from user *j*, the probability that user *i* will follow user *j* increases. We also investigate the probability that user *i* will follow user *j* when the number of users who are followed by user *i* and follow user *j* is exactly *c* (Fig 4). Fig 4 shows that there is a positive correlation between the number of common neighbors and the probability of following. This result supports the assumption of neighborhood-based link prediction that as the number of users followed by user *i* and following user *j* increases, the probability that user *i* will follow user *j* increases.

### Effectiveness of single link prediction technique

**Fig 5:**
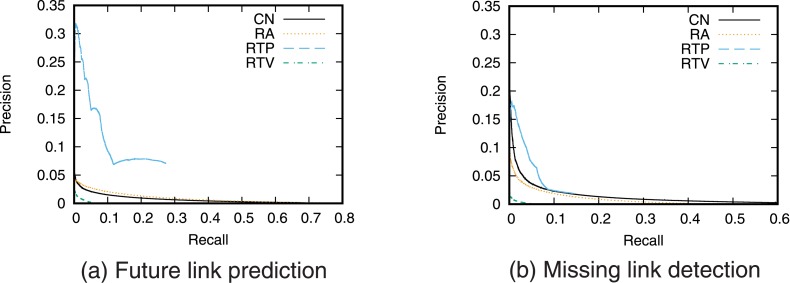
Precision-recall curve when using a single link prediction score.

**Fig 6:**
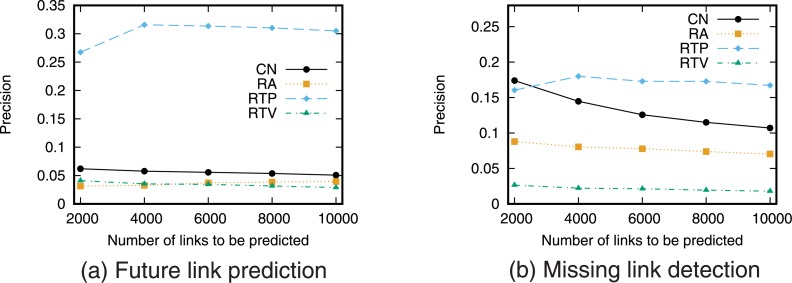
Precision when using a single link prediction score. The number of links to be predicted is fixed.

Next, we evaluate the prediction accuracy for the case of using a single link prediction technique. Fig 5 shows the precision–recall curve for the case of prediction using each link prediction technique. We then find the relationship between precision and recall by varying the threshold value *T*. We also calculated precision when the number of links to be predicted is fixed (Fig 6).

The precision of RTP, which is a link prediction technique based on the number of retweet posts, is higher than that of the other techniques for both future link prediction and missing link detection. This result supports the hypothesis that “as user *i* retweets more tweets from user *j*, the probability that user *i* will follow user *j* increases.” This also indicates that retweet history is useful information for link prediction on Twitter. In particular, when the recall is low, that is, when the threshold *T* is large, RTP gives higher precision than prediction techniques based on network topology. However, we can also see that since link prediction scores are assigned in RTP only when retweets are performed directly between users, high recall cannot be obtained even when the precision is low, that is, when the threshold *T* is small. Furthermore, the prediction accuracy of RTV, which is a link prediction technique based on the number of retweet views, is lower than that of the prediction techniques based on network topology. This is also consistent with existing research results [25].

Furthermore, comparing the prediction accuracy between future link prediction and missing link detection shows that the prediction accuracy of RTP in particular is high in the future link prediction task. This is thought to reflect the causal relationship between retweeting and link creation in which new links are created by the information flow generated by retweets [22, 23].

Note that the values of precision and recall obtained in this study are not high. One of the causes of low prediction accuracy is the so-called *class imbalance* problem [31, 34]. In the case of future link prediction, the number of positive cases (i.e., the number of newly formed links) is only 259,212, whereas the number of negative cases (the number of node pairs that do not form links) is 10,677,341,807. This is a common problem when performing link prediction in large-scale networks, and so the precision and recall of link prediction in large-scale networks tend to be low [34]. Pujari *et al.* discuss that filtering the prediction results by using community structure may be an effective approach to overcome the problem [31]. Using such an approach is necessary to achieve more accurate prediction in large-scale networks.

### Effectiveness of simple combination of multiple techniques

Next, we investigated the prediction accuracy for the case of using a simple combination of multiple link prediction techniques. We show the results for the case where RTP, which had the highest accuracy among predictions based on retweets, is combined with the network-based RA prediction. Note that results for other combinations are shown in supporting information (S1, S2, S3 and S4 Figs). We do this by using *l*_*RA*_(*i*, *j*) for *l*_*A*_(*i*, *j*) and *l*_*RTP*_ (*i*, *j*) for *l*_*B*_(*i*, *j*) in Eq (8). Although RTP is useful particularly when the threshold *T* is high, it cannot produce high recall even when *T* is lowered. In contrast, the network-based RA prediction produces high recall when the threshold *T* is lower. It is therefore expected that the link prediction accuracy can be increased by using a combination of these techniques.

**Fig 7:**
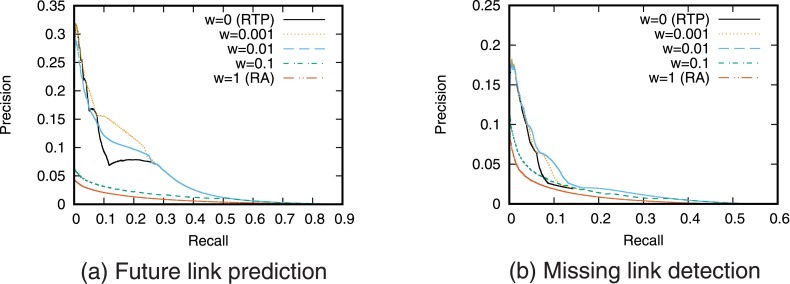
Precision-recall curve when combining RA and RTP.

**Fig 8:**
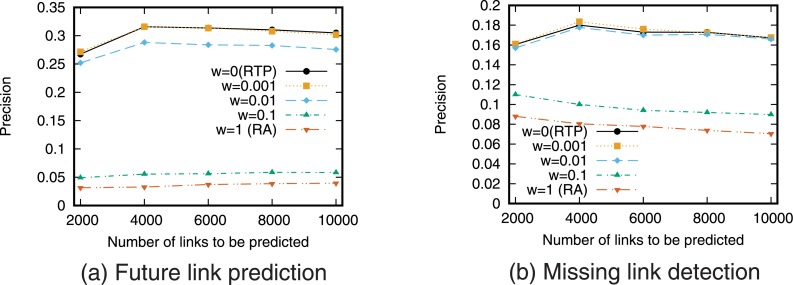
Precision when combining RA and RTP. The number of links to be predicted is fixed.

Fig 7 shows the precision-recall curve for the combination of the RTP prediction based on retweets and the RA prediction based on network topology. Fig 8 shows precision when fixed numbers of links are predicted. Note that the results with a weighting parameter of *w* = 0 are equivalent to the case of using RTP alone, and the results with *w* = 1 are equivalent to the case of using RA alone.

Fig 7 shows that the link prediction accuracy can be improved compared with the case of using only a single prediction technique by using a combination of prediction techniques based on retweets and network topology. Focusing on the weighting parameter *w*, we see that the prediction accuracy is highest when *w* = 0.001 for future link prediction and *w* = 0.01 for missing link detection. This indicates that a somewhat high weighting of the prediction score based on retweets is important for high prediction accuracy. In contrast, Fig 8 shows that the precision when combining RA and RTP is not higher than that when using RTP alone. This suggests that simply combining RA and RTP is not an effective means of predicting a small number of links.

**Fig 9:**
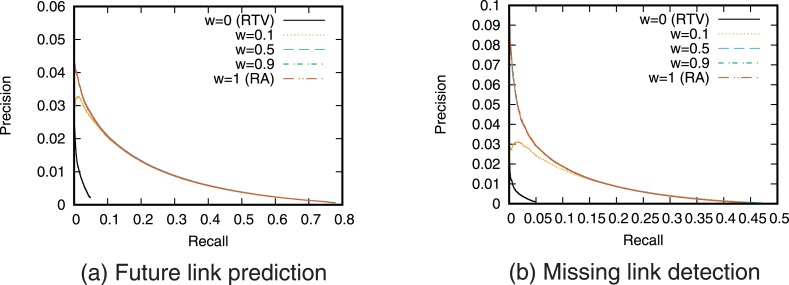
Precision-recall curve when combining RA and RTV.

**Fig 10:**
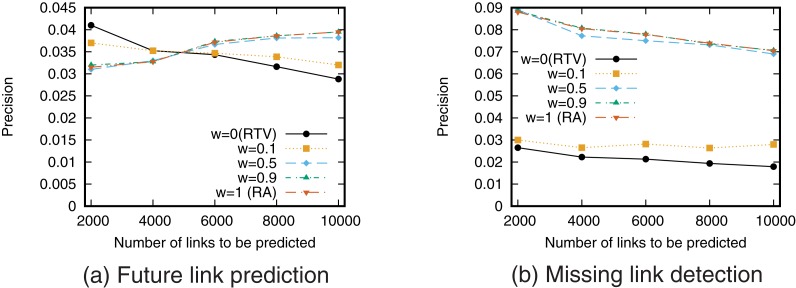
Precision when combining RA and RTV. The number of links to be predicted is fixed.

We next examine the combination of RTV and RA (Figs 9 and 10). We do this by using *l*_*RA*_(*i*, *j*) for *l*_*A*_(*i*, *j*) and *l*_*RTV*_(*i*, *j*) for *l*_*B*_(*i*, *j*) in Eq (8). From the results, we find that the combination of RTV and RA has almost no effect in terms of improving the prediction accuracy. In the case of a combination of RTP and RA, since RTP is effective when the recall is low, whereas RA is effective when the recall is high, the combination can cover the complementary weaknesses of the techniques. However, the accuracies of RTV and RA are similar. Therefore, the simple combination cannot improve the prediction accuracy.

### Effectiveness of rank aggregation

We next investigate the effectiveness of rank aggregation. We show the results for the case where RTP and RA are aggregated. The weight *β*_RA_ is changed and *β*_RTP_ is fixed to 1 − *β*_RA_.

**Fig 11:**
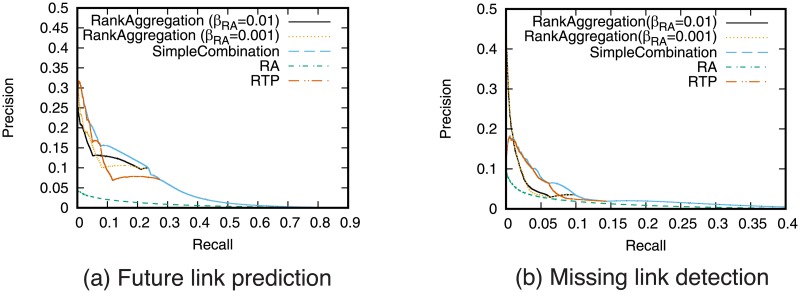
Precision-recall when combining RA and RTP using rank aggregation. In simple combination, RA and RTP are combined, and *w* = 0.001 is used for the weight of RA.

**Fig 12:**
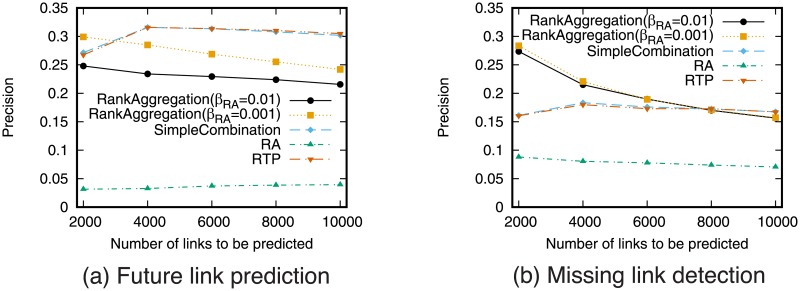
Precision when combining RA and RTP using rank aggregation. The number of links to be predicted is fixed. In simple combination, RA and RTP are combined, and *w* = 0.001 is used for the weight of RA.

Fig 11 shows the precision-recall curve for the rank aggregation of the RTP and the RA prediction. Fig 12 shows precision when fixed numbers of links are predicted. For comparison purposes, the results of simple combination of RTP and RA are also shown in these figures. The results for other combinations are shown in supporting information (S5, S6, S7 and S8 Figs).

These results show that link prediction accuracy can be improved using rank aggregation compared with the case of using only a single prediction technique particularly for the missing link detection task. Fig 12(b) shows that rank aggregation outperforms simple combination when fixed small number of links are predicted. While simple combination does not outperform RTP alone, rank aggregation can outperform RTP. This confirms that combining topology-based measure and retweet-based measure is an effective approach.

### Additional analyses

**Fig 13:**
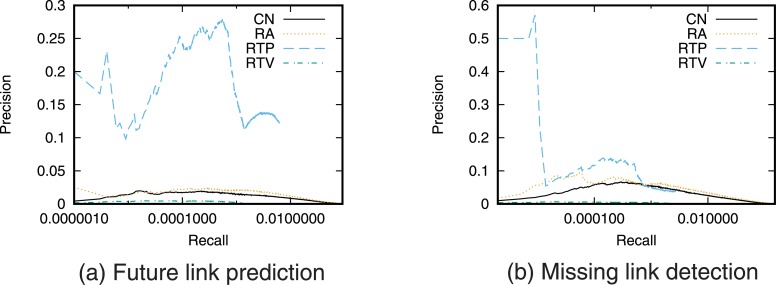
Precision–recall curve for less active users. Note that x-axis is log-scale.

**Fig 14:**
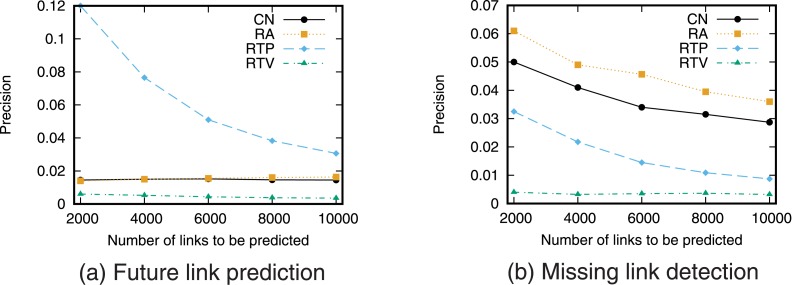
Precision for less active users. The number of links to be predicted is fixed.

For examining the applicability of retweet-based link prediction techniques, we investigate the prediction accuracy of each technique for less active users. From the target 356,453 users, we intentionally extract users whose frequency of retweeting is relatively low. We extracted users who performed retweeting less than or equal to 20 times during the period from January 1, 2014, to January 31, 2014. We then obtained 24,632 users. We examine the prediction accuracy of each technique for the 24,632 less active users (Figs 13 and 14).

These results show that when predicting a small number of future links, RTP achieves higher accuracy than other techniques. However, for the missing link detection task, topology-based techniques achieve higher accuracy than RTP. These results suggest that retweet-based technique, RTP is particularly effective for highly active users, and its effectiveness can be degraded for less active users. Note that prediction accuracy of topology-based methods is also not so high for the less active users. This suggests that predicting links of less active users is difficult task both for retweet-based and topology-based techniques.

We finally examine the correlation between link prediction techniques. Following [1], we counted the number of common predictions between two link prediction techniques. Table 3 shows the number of common predictions between two link prediction techniques when 2,000 links are predicted. Table 4 shows the number of *correct* common predictions. Both results are obtained from the future link prediction task.

**Table 3:** The number of common predictions obtained from different link prediction techniques when 2,000 links are predicted. (Future link prediction).

	CN	RA	RTV	RTP
CN	2000	750	49	3
RA		2000	31	2
RTV			2000	6
RTP				2000

**Table 4:** The number of common *correct* predictions obtained from different link prediction techniques when 2,000 links are predicted. The diagonal entries show the number of correct predictions for each technique (Future link prediction).

	CN	RA	RTV	RTP
CN	124	38	3	1
RA		63	1	0
RTV			82	2
RTP				535

These results show that retweet-based techniques and topology-based techniques make different predictions. If two link prediction scores are uncorrelated, the two uncorrelated scores can be effectively used in rank aggregation techniques [35] or supervised learning [8, 26, 27] for improving prediction accuracy. Therefore, this result suggests that using both retweet-based methods and topology-based methods is an effective approach for improving link prediction accuracy.

## Discussion

The results of this study indicate that in social media, link prediction based on retweet history is more effective than conventional prediction based on network topology alone for actively retweeting users. Furthermore, RTP prediction based on retweeted posts was more effective than RTV prediction based on retweet views. This suggests that the active behavior of posting a retweet indicates stronger user interest than does the passive behavior of viewing a retweet. Previous research on link prediction based on retweet history has been based on retweet views. The main contribution of this paper is that it shows the effectiveness of link prediction based on retweet posts.

We also evaluated the effectiveness of link prediction techniques for Japanese Twitter users. Previous research on link prediction based on retweets has targeted social media users in the United States and China. The results here show that link prediction based on retweets is effective for users in Japan, too. Although it is known that the behavior of social media users differs among languages and among cultures [36], from the results of this study, it can be anticipated that link prediction based on retweets is effective regardless of culture or language.

There are several limits to our analysis. Since this study intentionally covered only active users who retweet somewhat frequently, it is not possible to determine how effective link prediction based on retweets is for users who are less active. The number of users who retweeted at least once during the period of December 11–17, 2013 in our dataset was 4,175,906. The number of Twitter users in Japan as of 2013 was not reported, but was estimated to be approximately 16.6 million [37]. Since RTP cannot be applied to users who do not retweet, we can estimate that RTP cannot be applied to 75% of users. Moreover, the target users in our experiment retweeted at least 10 times in a week. The number of such users (i.e., users who retweeted at least 10 times during the period) in our dataset was 1,056,293. Therefore, the target users in our experiments are considered to be the top 6% of active users in terms of retweeting in Japan. Although our experimental results indicate that RTP is effective for such active users, the effectiveness of RTP is suggested to be degraded for less active users. As discussed in Ref. [22], it is known that among social media users with a small number of followers who are not particularly active, link creation occurs in accordance with network topology, and as the number of followers increases and the user becomes more active, link creation becomes based more on information flow through retweets. Ref. [22] and our results (Figs 13 and 14) suggest that prediction based on network topology is more effective for users who are not highly active. An important topic for future investigation is the relationship between user characteristics and effective link prediction techniques.

For the link prediction techniques based on network topology, we used only the popular neighborhood-based techniques, CN and RA. However, there exist other types of link prediction techniques based on network topology, including the Katz index [38], rooted PageRank [1], and PropFlow [39], which are path-based link prediction techniques. Comparing the prediction accuracy of these techniques with that of retweet-based techniques and combining these techniques with retweet-based techniques are important topics for future work.

Moreover, this study evaluated the effectiveness of link prediction by unsupervised approaches in particular. The link prediction scores we examined, such as RTP and RTV, can be used as features in supervised link prediction. The extent to which retweet history is useful in supervised link prediction should be investigated in the future.

## Conclusion

In this study, we investigated the effectiveness of user records of retweets for link prediction in the popular social media platform Twitter. Through extensive experiments, we found that using the records of retweets is an effective approach for link prediction on Twitter. Our experimental results showed that a link prediction technique based on retweet posts achieves better prediction accuracy than do popular topology-based techniques (specifically, CN and RA) or techniques based on retweet views for actively retweeting users. Our results also showed that the accuracy of link prediction can be increased by combining retweet records and network topology.

## Supporting Information

S1 Fig Precision-recall curve when combining CN and RTP. (EPS)

S2 Fig Precision-recall curve when combining RTP and RTV. (EPS)

S3 Fig Precision when combining CN and RTP. The number of links to be predicted is fixed.
(EPS)

S4 Fig Precision when combining RTP and RTV. The number of links to be predicted is fixed.
(EPS)

S5 Fig Precision-recall curve when combining CN and RTP using rank aggregation. In simple combination, CN and RTP are combined, and *w* = 0.001 is used for the weight of CN.
(EPS)

S6 Fig Precision-recall curve when combining RTP and RTV using rank aggregation. In simple combination, RTV and RTP are combined, and *w* = 0.001 is used for the weight of RTV.
(EPS)

S7 Fig Precision when combining CN and RTP using rank aggregation. The number of links to be predicted is fixed. In simple combination, CN and RTP are combined, and *w* = 0.001 is used for the weight of CN.
(EPS)

S8 Fig Precision when combining RTP and RTV using rank aggregation. The number of links to be predicted is fixed. In simple combination, RTV and RTP are combined, and *w* = 0.001 is used for the weight of RTV.
(EPS)
